# The immunoregulatory protein CD200 as a potentially lucrative yet elusive target for cancer therapy

**DOI:** 10.18632/oncotarget.28354

**Published:** 2023-02-04

**Authors:** Anqi Shao, David M. Owens

**Affiliations:** ^1^Department of Dermatology, Columbia University Irving Medical Center, Vagelos College of Physicians and Surgeons, New York, NY 10032, USA; ^2^Department of Pathology and Cell Biology, Columbia University Irving Medical Center, Vagelos College of Physicians and Surgeons, New York, NY 10032, USA

**Keywords:** CD200 receptor, oncoimmunology, immunotherapy, tumor microenvironment, immune checkpoint inhibition

## Abstract

CD200 is an immunoregulatory cell surface ligand with proven pro-tumorigenic credentials via its ability to suppress CD200 receptor (CD200R)-expressing anti-tumor immune function. This definitive role for the CD200-CD200R axis in regulating an immunosuppressive tumor microenvironment has garnered increasing interest in CD200 as a candidate target for immune checkpoint inhibition therapy. However, while the CD200 blocking antibody samalizumab is still in the early stages of clinical testing, alternative mechanisms for the pro-tumorigenic role of CD200 have recently emerged that extend beyond direct suppression of anti-tumor T cell responses and, as such, may not be susceptible to CD200 antibody blockade. Herein, we will summarize the current understanding of CD200 expression and function in the tumor microenvironment as well as alternative strategies for potential neutralization of multiple CD200 mechanisms in human cancers.

## INTRODUCTION

CD200 is a type I membrane-associated glycoprotein and a member of the immunoglobin superfamily [1–3]. While initially reported in the rat thymus in 1979 as the antigen of the MRC OX2 antibody [4], CD200 expression has since been detected on a variety of cells of hematopoietic, such as macrophages, dendritic cells, B cells, and activated T cells, and non-hematopoietic origin, including neurons, endothelial cells, trophoblasts, and epithelial keratinocytes [4–10]. The receptor for CD200, CD200R, is expressed mainly in myeloid cells, but is also detected on lymphoid lineage cells, such as natural killer (NK) and T cells [11–14] and contains a cytoplasmic tail capable of initiating downstream signaling cascades [3]. As CD200 ligand contains a short cytoplasmic tail of 19 amino acid residues that lacks a consensus signaling motif [1, 3, 15], CD200 function is primarily mediated via cell-cell interaction-dependent engagement of the CD200-CD200R axis.

The CD200-CD200R axis primarily functions as an immunoregulatory signaling pathway. Mice lacking *Cd200* exhibit elevated numbers of activated Cd200r+ macrophages and granulocytes [11] but exhibit normal myelopoiesis [16]. Phenotypically, Cd200 loss and concomitant increased activated macrophage levels manifest in chronic nervous system inflammation, early onset of experimental autoimmune encephalomyelitis and susceptibility to experimental autoimmune uveoretinitis [11, 17]. These observations underscore an essential role of Cd200 in maintaining tissue homeostasis by tempering the level of activated myeloid cells. Under pathological conditions, Cd200 also plays a key role in directly suppressing Th1-mediated inflammation [18–20], thereby orchestrating a balance between effective pathogen clearance and preventing immunopathology. Collectively, these observations confer a primary immunosuppressive function for CD200 in maintaining tissue homeostasis that is facilitated via engagement with CD200R.

CD200 expression is reported across most cancer types including hematologic malignancies such as acute myeloid leukemia (AML) [21], multiple myeloma (MM) [22], chronic lymphocytic leukemia (CLL) [23], and B-cell lymphoma [24]; solid tumors such as rectal [25], breast [26–27], colon [28–29], lung [30–31], ovarian [32], head and neck [33–34], glioma [35], pancreatic [36–37], and bladder [38]; and a variety of skin cancers including squamous cell carcinoma [39–41], basal cell carcinoma [42], Merkel cell carcinoma [43] and melanoma [32, 44–45].

**Figure 1:**
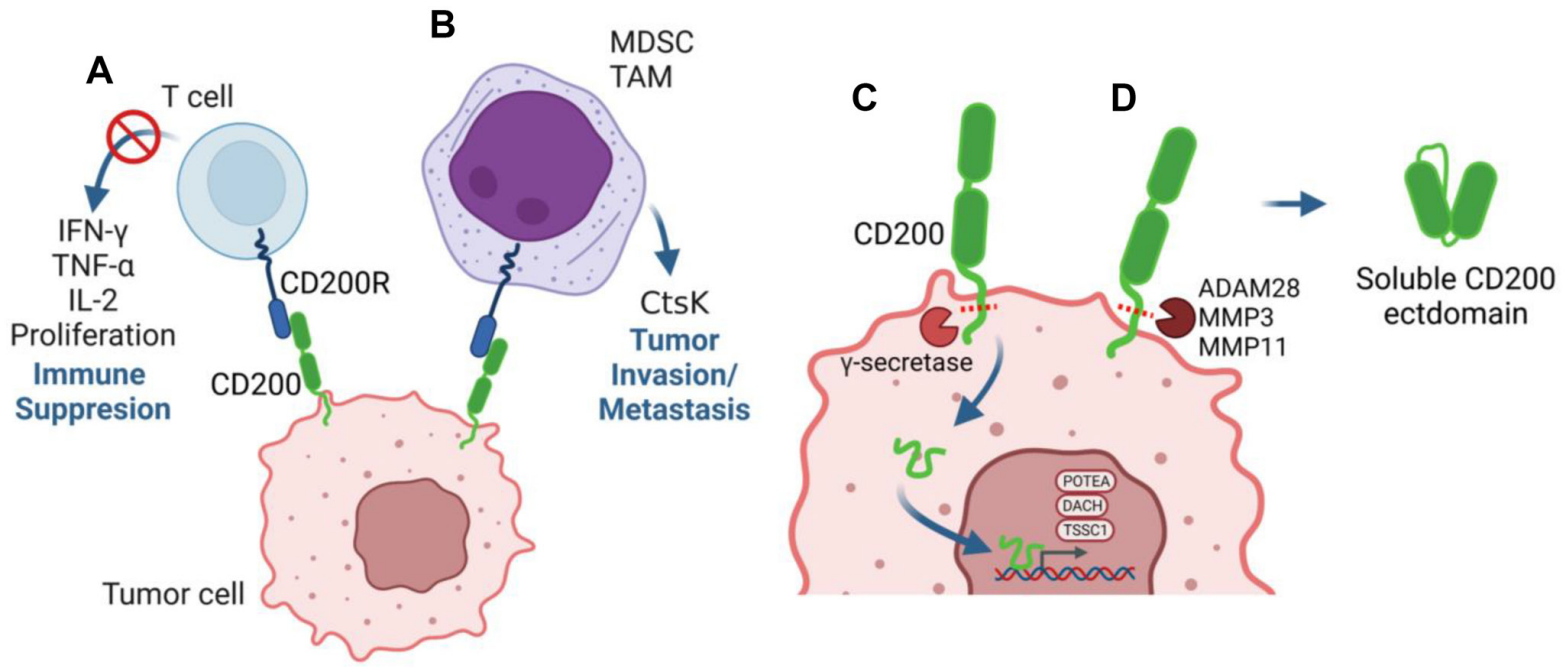
Multiple mechanisms underly the pro-tumorigenic role of CD200. (**A**, **B**) Tumor cell-extrinsic mechanisms for CD200 direct suppression of CD200R+ Th1 anti-tumor responses including pro-inflammatory cytokine production and proliferation (A) or CD200R-dependent activation of tumor-infiltrating myeloid lineages including MDSC and TAM (B). (**C**, **D**) Tumor cell-intrinsic mechanisms for CD200 include γ-secretase cleavage of the short CD200 cytoplasmic tail that translocates to the nucleus and regulates the expression of pro-tumorigenic target genes (C) or ADAM or MMP cleavage of CD200 to generate a soluble CD200 ectodomain (D), which has been shown to circulate systemically and in the tumor microenvironment. Abbreviations: MDSC: myeloid-derived suppressor cell; TAM: tumor-associated macrophage; ADAM: a disintegrin and metalloprotease domain; MMP: matrix metalloproteinase.

Similar to other immune checkpoint proteins, such as cytotoxic T lymphocyte antigen 4 (CTLA-4) and program death-1 (PD-1), CD200 is thought to play a pro-tumorigenic role, via engagement of CD200R, in many tumor types primarily by suppressing anti-tumor T-cell and natural killer cell responses [46–49] (Figure 1A). In melanoma cells, CD200 can inhibit Th1 cytokine production [32] and, in CLL, cytotoxic T cell proliferation [47]. CD200 overexpression in AML can directly suppress anti-tumor NK cell and memory T cell functions [48–49], and the suppression of pro-inflammatory cytokines by CD4+ Th1 cells and CD8+ memory T cell numbers in AML patients can be restored with anti-CD200 treatment [48–49]. Collectively, these studies illustrate a key pro-tumorigenic role for CD200 in directly suppressing T-cell and NK cell anti-tumor function and that blocking the engagement of the CD200-CD200R axis may provide therapeutic benefit to patients with CD200-expressing tumors. Further supporting this concept, in syngeneic and xenograft murine tumor models, treatment with CD200 neutralizing antibodies restored lymphocyte-mediated anti-tumor responses *in vivo* [26, 50].

A recently completed Phase I trial assessed treatment responses of CLL and MM patients to samalizumab, a humanized anti-CD200 monoclonal antibody designed to block CD200-CD200R binding while minimizing cytotoxicity to CD200-expressing immune cell subsets (by using an IgG region with limited effector function) [51]. Decreases in overall tumor burden were observed in approximately 65% of CLL patients; however, a durable response was only observed in one out of twenty-three patients (4%). MM patients were refractory to samalizumab and a range of mild to moderate adverse outcomes were reported in the majority of CLL and MM patients, including skin rashes, joint stiffness/pain, headaches, and blood disorders. These adverse outcomes are consistent with the autoimmune phenotype reported in *Cd200* null mice [11] and those observed in patients treated with PD-1 and/or CTLA-4 antibody therapy [52]. In 2017, a previous samalizumab Phase I trial was completed in patients with solid malignancies; however, to our knowledge the results of this study have yet to be disclosed. While the initial Phase I trial findings for samalizumab in CLL patients [51] certainly warrant further clinical investigation of different dosing regiments and its efficacy in additional blood cancers, shortcomings related to partial responses, lack of a durable response and the high incidence of mild to moderate toxicities raise concerns over the potential impact of CD200 antibody blockade therapy may hold for certain blood cancers other than CLL and solid malignancies.

A plausible explanation for the observed shortcomings in the samalizumab Phase I trial may be the alternative mechanisms for the CD200 pro-tumorigenic role that have recently emerged. Some of these alternative mechanisms extend beyond direct suppression of anti-tumor T cell responses and, as such, may not be susceptible to CD200 antibody blockade. First, in addition to targeting T-cell and NK cell numbers and/or activity, the recruitment and function of myeloid derived suppressor cells (MDSCs) and tumor-associated macrophages (TAMs) are known to be regulated by the CD200-CD200R axis in human cancer [37, 39, 43, 53] (Figure 1B). Initial work from our laboratory demonstrated MDSCs and TAMs constitute greater than 90% of the CD200R+ cells in the microenvironment of cutaneous squamous cell carcinoma (cSCC) and that production of critical microenvironment cues by these tumor-infiltrating myeloid lineages, including GM-CSF and G-CSF, was dependent on engagement of the Cd200-Cd200r axis [39]. More recently, using a *Cd200* conditional null mouse model, we identified the collagen peptidase Cathepsin K (Ctsk) as a crucial target gene of the Cd200-Cd200r signaling axis in Cd200r+ tumor-infiltrating myeloid lineages, which was required for tumor cell invasion and metastasis [54] (Figure 1B). Interestingly, CTSK is a known biomarker for a variety of cancer types [55] and its expression is associated with metastasis of human solid malignancies [56–57] suggesting a broader functional role for CTSK in human tumor metastasis.

Adding to this complexity, non-canonical mechanisms for CD200 in tumorigenesis have also recently emerged. The intracellular tail of CD200, previously thought to be signaling inert, is a target for cleavage by γ-secretase, resulting in release of a CD200 tail fragment that is capable of nuclear translocation and DNA binding [58]. DNA binding by the CD200 cytoplasmic tail leads to increased expression of transcription factors associated with leukemic cell growth [58] (Figure 1C). In the extracellular space, ADAM28-mediated CD200 ectodomain shedding leads to increased serum levels of a biologically-active, soluble CD200 ectodomain fragment in B-cell CLL patients [59] (Figure 1D). Recently, MMP-mediated CD200 ectodomain shedding in basal cell carcinoma was shown to regulate NK cell dysfunction and apoptosis in the microenvironment [60] (Figure 1D). It remains an open question as to whether antibody therapies designed to block membrane-tethered CD200-CD200R binding may be effective against soluble CD200 ectodomain fragments of variable sizes that may be generated by different protease families. Collectively, these observations underscore i) a broader pro-tumorigenic role for CD200 in the tumor microenvironment and ii) potential pleiotropic mechanisms adopted by CD200 to mediate tumor cell survival, invasion and metastasis that may be difficult to block via a monoclonal antibody treatment modality targeting a specific ectodomain region.

Overall, the inconsistent response rates across different tumor types, low incidence of durable responses and observed undesirable cytotoxicity outcomes to samalizumab therapy together with newly emerging non-canonical roles for CD200 in cancer provides rationale for alternative strategies designed to efficaciously target this protein. An underexplored aspect of the role of CD200 in cancer is the identification of tumor-specific mechanisms for the regulation of CD200 expression. A better understanding of CD200 regulatory mechanisms may be relevant for multiple CD200 pro-tumorigenic functions including engagement of the CD200-CD200R axis, transcriptional mechanisms related to the cleaved cytoplasmic tail and ectodomain shedding. Clouding our understanding of this issue is that, in normal and neoplastic cells, the induction or suppression of CD200 expression is reported to be associated with a wide variety of signaling pathways and CD200 expression can be regulated by both constitutive and inducible pathways. Genomic analysis of the human CD200 promoter identified C/EBPβ as a key regulator of constitutive CD200 expression [61]; while upstream enhancer regions harbor binding sites for IFNγ- and TNFα-induced signaling pathway effectors that are proposed to be necessary for inducible CD200 expression [62].

In the central nervous system (CNS), the CD200-CD200R axis is a fundamental facilitator of neuron-microglia cell-cell interactions that maintain physiological levels of inflammation. Multiple pathways are reported to regulate CD200 expression in the CNS. In both astrocytes [63] and neurons [64], CD200 expression is regulated by FGFR1 activation, which is critical for suppression of neuroinflammation. In addition to FGFR signaling, treatment with PPARγ ligands can suppress CD200 induction in activated glial cells [65], which is thought to play a functional role in PPARγ-mediated neuroprotection. Under pathological conditions, CD200 expression in microglia can be inhibited by IFNγ derived from a leaky blood brain barrier [66]. In other tissues, the molecules involved in the regulation of CD200 expression appear to be unique to each cell lineage. CD200 is a downstream target of p53 during caspase-dependent dendritic cell apoptosis [67]. In a mouse model of meningococcal infection, CD200 induction in macrophages is dependent on TLR4 and downstream NF-κB signaling [68]. In bone marrow mesenchymal stem cells, CD200 expression can be induced by osteogenic and pro-inflammatory cytokines also in a NF-κB-dependent manner [69]. In the skeletal system, CD200 expression in osteoblasts is dependent on IL15RA signaling [70]. In the lung, both airway epithelial cell and capillary endothelial cell expression of CD200 is shown to be regulated by corticosteroids [71–72]. In hair follicle epithelial progenitors, CD200 expression is reported to be dependent on β1 integrin activation [73].

In human cancers, deeper knowledge gaps exist in our understanding of the regulation of CD200 expression. First, relative to normal tissue, there is a paucity of information regarding CD200 expression regulation in human cancers. Second, CD200 expression may be present at early stages of development for certain tumors, whereas, in other lesions, CD200 expression is induced during late-stage progression and only in a sub-set of tumor cells. Third, there appears to be little to no overlap in the putative molecular regulators of CD200 expression across different tumor types. Currently, these bottlenecks preclude our ability to effectively pinpoint potential regulatory targets to block CD200 expression. In metastatic melanoma, CD200 is regulated by ERK activation downstream of N-RAS or B-RAF mutations [44] suggesting that CD200 induction is an early event in melanoma pathogenesis. In human and murine cSCC lesions, we previously observed little to no CD200 expression in early, well-differentiated tumors [39]. However, CD200 expression was induced in poorly-differentiated primary and metastatic cSCC with enrichment of CD200 localized to leading edge tumor cells [39, 54]. In endometriosis patients, CD200 expression is upregulated in lesional stromal cells and in the blood and 17β-estradiol treatment of stromal cells in culture increased expression of CD200 [74]. In colorectal carcinoma cells, CD200 expression is dependent on the activity of the Rho GTPase effector protein FMNL2 [75]. CD200 is a target of miR-499a and a polymorphism in miR-499a is a poor prognostic factor for non-small cell lung cancer cases who also exhibit elevated CD200 expression [76].

In the future, unbiased genomic- and proteomic-based approaches may help to clarify these issues by identifying tumor-specific mechanisms of CD200 expression regulation across a variety of human cancers that may be leveraged for broader therapeutic benefit.
